# Identification and Characterization of Phenylpropanoid Biosynthetic Genes and Their Accumulation in Bitter Melon (*Momordica charantia*)

**DOI:** 10.3390/molecules23020469

**Published:** 2018-02-21

**Authors:** Do Manh Cuong, Soon-Jae Kwon, Jin Jeon, Yun Ji Park, Jong Seok Park, Sang Un Park

**Affiliations:** 1 Department of Crop Science, Chungnam National University, 99 Daehak-ro, Yuseong-gu, Daejeon 34134, Korea; domanhcuong87hy@gmail.com (D.M.C.); jeonjin519@gmail.com (J.J.); yunji0825@hanmail.net (Y.J.P.); 2 Korea Atomic Energy Research Institute, Advanced Radiation Technology Institute, 29 Geumgu-gil, Jeongeup-si, Jeollabuk-do 56212, Korea; soonjaekwon@kaeri.re.kr; 3 Department of Horticulture, Chungnam National University, 99 Daehak-ro, Yuseong-gu, Daejeon 34134, Korea; jongseok@cnu.ac.kr

**Keywords:** bitter melon, *Momordica charantia*, phenylpropanoid, flavonoid, LED

## Abstract

Phenylpropanoids and flavonoids belong to a large group of secondary metabolites, and are considered to have antioxidant activity, which protects the cells against biotic and abiotic stresses. However, the accumulation of phenylpropanoids and flavonoids in bitter melon has rarely been studied. Here, we identify ten putative phenylpropanoid and flavonoid biosynthetic genes in bitter melon. Most genes were highly expressed in leaves and/or flowers. HPLC analysis showed that rutin and epicatechin were the most abundant compounds in bitter melon. Rutin content was the highest in leaves, whereas epicatechin was highly accumulated in flowers and fruits. The accumulation patterns of *trans*-cinnamic acid, *p*-coumaric acid, ferulic acid, kaempferol, and rutin coincide with the expression patterns of *McPAL, McC4H, McCOMT, McFLS,* and *Mc3GT*, respectively, suggesting that these genes play important roles in phenylpropanoid and flavonoid biosynthesis in bitter melon. In addition, we also investigated the optimum light conditions for enhancing phenylpropanoid and flavonoid biosynthesis and found that blue light was the most effective wavelength for enhanced accumulation of phenylpropanoids and flavonoids in bitter melon.

## 1. Introduction

**Figure 1:**
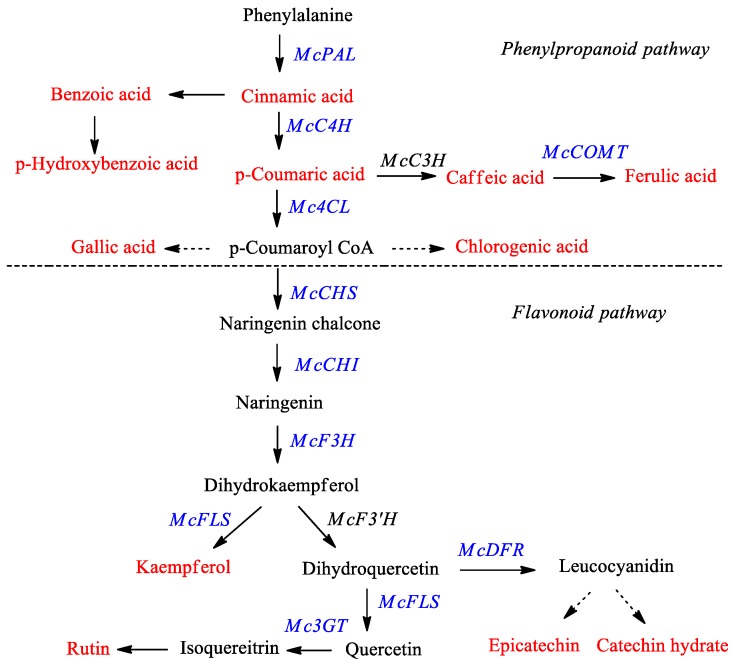
Proposed phenylpropanoid and flavonoid biosynthetic pathway in bitter melon. The red color denotes the phenylpropanoids and flavonoids measured in this study by HPLC analysis and the blue color indicates genes monitored via real time-PCR. PAL—phenylalanine ammonia-lyase; C4H—cinnamate 4-hydroxylase; C3H—coumarate 3-hydroxylase; COMT—caffeic acid 3-*O*-methyltransferase; 4CL—4-coumaroyl CoA ligase; CHS—chalcone synthase; CHI—chalcone isomerase; F3H—flavanone 3-hydroxylase; F3′H—flavonoid 3′-hydroxylase; FLS—flavonol synthase; DFR—dihydroflavonol-4 reductase; 3GT—flavonoid 3-*O*-glucosyltransferase.

*Momordica charantia*, commonly known as “bitter melon” or “bitter gourd”, is a medicinal plant belonging to family Cucurbitaceae. Traditionally, it has been used to treat diseases such as HIV, diabetes, cancer, arthritis, liver disorders, and gastric problems [1,2,3,4,5,6]. Researchers have found that the important components of bitter melon with respect to its pharmaceutical application are phenolic acids, flavonoids, triterpenes, and carotenoids [7]. Phenolic compounds (such as phenylpropanoids and flavonoids) belong to the largest group of secondary metabolites produced by plants, and are known to have antioxidant activity, protecting the cells against biotic and abiotic stresses including infections, wounding, UV irradiation, exposure to ozone, pollutants, and herbivores [8,9,10]. Considering these biological characteristics and functions, phenylpropanoids and/or flavonoids have been studied in many plants such as *Fagopyrum esculentum* [11,12], *Fagopyrum tartaricum* [13,14,15], *Fragaria ananassa* Duch. [16], *Morus alba* L. [17], *Scutellaria baicalensis* [18], *Scutellaria lateriflora* [18], and *Astragalus membranaceus* [19,20]. The phenolic compound content and antioxidant properties of bitter melon were described previously by Horax et al. [21]. However, little is known about the genes involved in phenylpropanoid and flavonoid biosynthesis pathways in bitter melon. Phenylpropanoids and flavonoids belong to a large class of plant phenols that are produced through the shikimic acid pathway. The biosynthesis of phenylpropanoids and flavonoids and the various catalyzing enzymes involved are shown in Figure 1.

Recently, irradiation by different wavelengths of light-emitting diodes (LEDs) is being used as source of artificial lighting in plant production systems and is attracting much attention due to its high potential for commercial applications. LEDs have many advantages such as small size, long operating lifetime, wavelength specificity, low heat emission, high energy-conversion efficiency, and adjustable light intensity [22,23]. The use of LEDs has also allowed researchers to easily analyze the effects of different wavelengths and light intensities on the accumulation of secondary compounds in controlled environments. Irradiation by different wavelengths of light with light-emitting diodes (LEDs) has been reported to efficiently modulate phenolic and flavonoid biosynthesis in plants such as lettuce [24,25], buckwheat [26,27], and Chinese cabbage [28]. However, it has not been reported in bitter melon.

In this study, we identified the genes involved in phenylpropanoid and flavonoid synthesis in bitter melon, using a transcriptome database reported in a previous study [29]. We also analyzed the expression profiles of these genes and the accumulation of phenylpropanoids and flavonoids in different organs and plantlets to investigate the relationship between phenylpropanoid and flavonoid contents and their gene expression levels. In addition, we investigated whether white, blue, and red light conditions could enhance phenylpropanoid and flavonoid accumulation in bitter melon. To the best of our knowledge, the present study is the first to apply transcriptome sequence analysis in the study of phenylpropanoid and flavonoid gene expression in bitter melon. It marks a first step towards possible bioengineering of bitter melon to increase the yield of these important phenylpropanoids and flavonoids.

## 2. Results and Discussion

### 2.1. Expression of Phenylpropanoid and Flavonoid Genes in Different Organs of Bitter Melon

A previous study showed that approximately 88,703 unigenes have been identified in bitter melon seedlings [29]. Among the unigenes from the bitter melon database, we could identify 10 candidate genes (*McPAL, McC4H, Mc4CL, McCOMT, McCHS, McCHI, McF3H, McFLS, McDFR,* and *Mc3GT*) that encode enzymes related to phenylpropanoid and flavonoid biosynthetic pathways (Table 1). Their homology was confirmed by blastp and designed as follows: *McPAL* (416 amino acids (aa)), *McC4H* (502 aa), *Mc4CL* (556 aa), *McCOMT* (369 aa), *McCHS* (402 aa), *McCHI* (252 aa), *McF3H* (370 aa), *McFLS* (335 aa), *McDFR* (310 aa), and *Mc3GT* (462 aa) (Table 1). These genes were highly similar to those in other species belonging to the Cucurbitaceae, such as *Cucumis sativus* and *Cucumis melo*.

**Figure 2:**
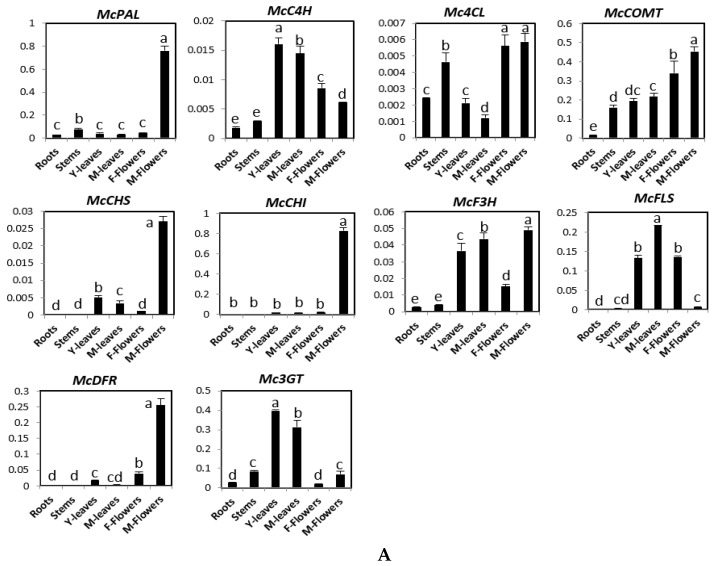

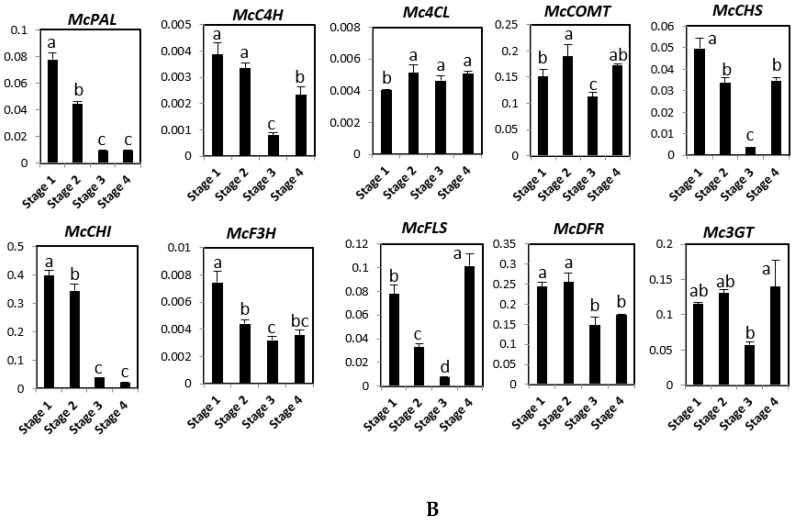
Expression levels of genes involved in phenylpropanoid and flavonoid biosynthesis pathway in different organs (**A**) and four fruit stages (stage 1–4) (**B**) of bitter melon plants. The vertical axes show the expression relative to *cyclophilin* and the height of each bar and the error bars indicate the mean and standard error, respectively, from three independent measurements. Y-leaves: young leave; M-leaves: mature leaves; F-flowers: female flowers; M-flowers: male flowers. Letters a–e indicate significant differences (*p* < 0.05).

The expression patterns of these genes were analyzed in roots, stems, young leaves, mature leaves, male flowers, female flowers, and four fruit developmental stages using quantitative RT-PCR (Figure 2). Figure 2A shows that *McPAL, McCHS, McCHI,* and *McDFR* were mainly expressed in male flowers. The expression level of *McC4H* was the highest in young and mature leaves, followed by female flowers and male flowers. Although the level of *Mc4CL* expression was found to be similarly high in stems, male flowers, and female flowers, it was low in the roots and leaves. *McF3H* had a higher expression in male flowers, young leaves, and mature leaves than in other organs. *McFLS* showed the highest levels of expression in mature leaves, followed by young leaves and female flowers, and low expression in roots, stems, and male flowers. *McCOMT* was highly expressed in both male and female flowers, intermediately expressed in stems, young leaves, and mature leaves, and expressed at low levels in the roots. *Mc3GT* was expressed at high levels in leaves than in other organs. During fruit ripening, the expression of *McPAL*, *McCHI*, *McF3H*, and *McDFR* declined gradually (Figure 2B). Taken together, while the expression of *McC4H*, *McCHS*, and *McFLS* declined from stage 1 to stage 3 and increased in stage 4, the expression of *Mc4CL, McCOMT*, and *MC3GT* increased from stage 1 to stage 2, but it declined in stage 3 before increasing again in stage 4 after fruit development (Figure 2B).

### 2.2. Phenylpropanoid and Flavonoid Accumulation in Different Organs of Bitter Melon

Quantification of phenylpropanoid and flavonoid content is shown in Table 2 and Table 3. We found that phenylpropanoids and flavonoids were rarely biosynthesized in the roots, whereas abundant phenylpropanoids and flavonoids were detected in the leaves and flowers of bitter melon (Table 2). The observed distribution of phenylpropanoids and flavonoids was similar to that observed in other plants such as *L. chinense* [30] and *Fagopyrum esculentum* [31]. The accumulation of 4-hydroxybenzoic acid, caffeic acid, *p*-coumaric acid, and rutin was more concentrated in the leaves than in the other organs, whereas benzoic acid was more abundant in the stems than in the other organs. Rutin was the most abundant component in young and mature leaves (3970.83 and 2277.93 μg/g dry weight, respectively), with concentrations 107- and 61-fold than that in the root (37.035 μg/g dry weight), respectively (Table 2). Rutin content has also been reported to be the highest in leaves of *Morus alba L.* [17] and *Lycium chinense* [30]. The accumulation of gallic acid, catechin hydrate, epicatechin, and trans-cinnamic acid was only found in the flowers. Among phenolic compounds, epicatechin was the most abundant component in female and male flowers (568.67 and 660.35 μg/g dry weight, respectively). The content of chlorogenic acid was considerably higher in male flowers (58.7 μg/g dry weight) and female flowers (49.39 μg/g dry weight) that in the other organs. Ferulic acid content was the highest in female flowers (9.49 μg/g dry weight) and male flowers (10.27 μg/g dry weight), whereas only small amounts were found in stems, young leaves, and mature leaves.

Phenylpropanoid and flavonoid content on the developed fruits was similar to that in the four developmental stages (Table 3). The amount of the phenolic compounds varied highly from stage 1 (green color) to stage 4 (orange color). For example, epicatechin and *p*-coumaric acid were more concentrated in stage 2 than in the other fruit stages. Caffeic acid accumulated only at the first stage of fruit maturation (being 13.91 μg/g dry weight), while kaempferol accumulated only at stages 1 and 4. The accumulation of rutin increased from stage 1 (12.49 μg/g dry weight) to stage 3 (20.7 μg/g dry weight), and then decreased again at stage 4 (11.15 μg/g dry weight). The chlorogenic acid content increased from stage 1 (37.89 μg/g dry weight) to stage 4 (40.47 μg/g dry weight). Similar contents of ferulic acid were found in the four fruit stages (Table 3). 

### 2.3. Expression of Phenylpropanoid and Flavonoid Genes in Different Developmental Stages of Bitter Melon Plantlets

**Figure 3:**
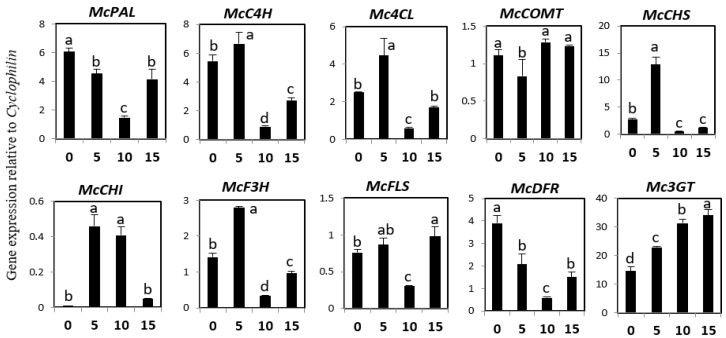
Expression levels of phenylpropanoid and flavonoid biosynthetic genes in different developmental stages of bitter melon plantlets. The height of each bar and the error bars indicate the mean and standard error, respectively, from three independent measurements. The vertical axes show the expression relative to *cyclophilin*. Units on the horizontal axes indicate the days after sowing. Letters a–d indicate significant differences (*p* < 0.05).

Expression analysis for the phenylpropanoid and flavonoid genes was carried out at different developmental stages (0 days after sowing (DAS), 5 DAS, 10 DAS, and 15 DAS) using real-time PCR (Figure 3). The expression of phenylalanine ammonia-lyase (*McPAL*) and dihydroflavonol 4-reductase *(McDFR*) was the highest at 0 DAS, intermediate at 5 and 15 DAS, and the lowest at 10 DAS. The expression patterns of *McC4H, Mc4CL, McCHS,* and McF3H were similar, with slightly more expression observable at 5 DAS than at other developmental stages. *McCHI* showed higher expression levels at 5 and 10 DAS, and lower levels at 0 and 15 DAS. *McFLS* expression was the highest at 0, 5, and 15 DAS, and the lowest at 10 DAS. The expression level of *Mc3GT* increased from 0 to 5 to 10 DAS, reaching its highest level at 15 DAS.

### 2.4. Phenylpropanoid and Flavonoid Accumulation in Different Developmental Stages of Bitter Melon Plantlets

HPLC analysis of bitter melon plantlets in different developmental stage identified nine phenylpropanoid and flavonoid components, including caffeic acid, *trans*-cinnamic acid, ferulic acid, benzoic acid, 4-hydroxybenzoic acid, and chlorogenic acid, *p*-coumaric acid, catechin hydrate, and rutin, as shown in Table 4. The accumulated amounts ranged from undetectable to 6.79, 4.91 to 5.29, 0.58 to 5.14, 0.66 to 4.61, and 4.14 to 7.92 μg/g dry weight for caffeic acid, ferulic acid, *p*-coumaric acid, *trans*-cinnamic acid, and benzoic acid, respectively. The accumulation levels of chlorogenic acid at 0, 10, and 15 DAS were similar, but it was not detectable at 5 DAS. The level of 4-hydroxybenzoic acid was the highest at 10 DAS (40.98 μg/g dry weight), followed by the levels at 15 DAS (18.82 µg/g dry weight) and 0 DAS (0.375 μg/g dry weight), but it was undetectable at 5 DAS. High amounts of rutin were found at 10 and 15 DAS (157.935 and 208.97 μg/g dry weight, respectively), whereas lower amounts were found at 0 DAS (12.27 μg/g dry weight). Finally, high amounts of catechin hydrate were found at 0 and 5 DAS (26.52 and 28.875 μg/g dry weight, respectively), and low amounts were found at 15 DAS (13.12 μg/g dry weight).

The expression levels of *Mc3GT* increased from 0 to 15 DAS, corresponding to the rutin accumulation levels. *McDFR* expression was high at 0 and 5 DAS, but low at 10 and 15 DAS, corresponding to the catechinhydrate levels. Similarly, the expression of *McCOMT* was found to correspond with ferulic acid content. We found that the expression levels of *Mc4CL, McCHS, McCHI, McF3H,* and *McFLS* increased from 0 to 5 DAS, but then decreased at 10 DAS before increasing again at 15 DAS. However, the content of phenylpropanoid and flavonoid did not change correspondingly. These results suggest *McPAL, McC4H, Mc3GT, McDFR,* and *McCOMT* might be intimately related to the phenylpropanoid and flavonoid content in bitter melon plantlets.

### 2.5. Effects of White, Blue, and Red Lights on the Expression of Phenylpropanoid and Flavonoid Genes

**Figure 4:**
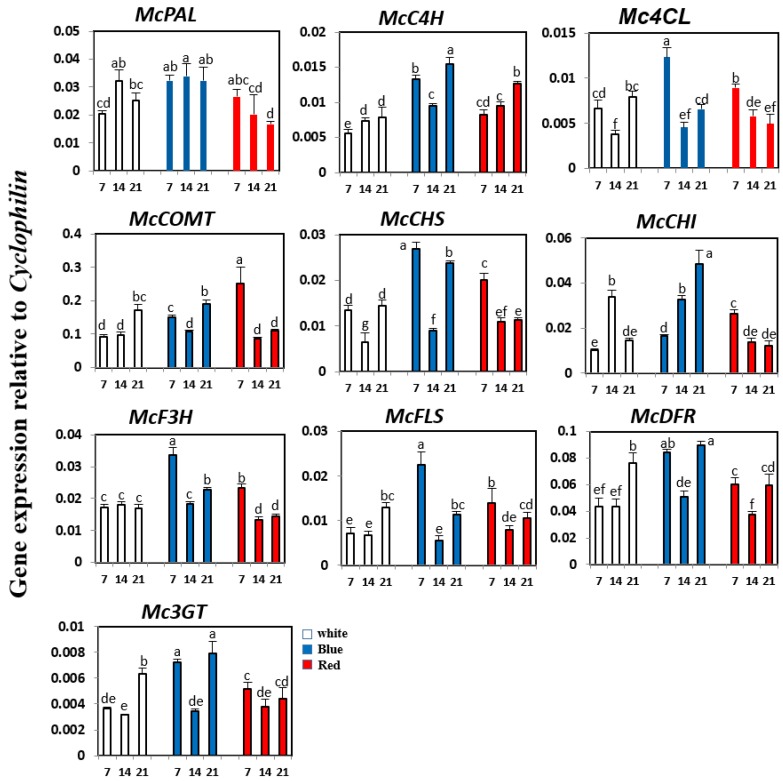
Expression levels of phenylpropanoid and flavonoid biosynthetic genes in bitter melon exposed to white, blue, and red LED. The height of each bar and the error bars indicate the mean and standard error, respectively, from three independent measurements. The vertical axes show the expression relative to *cyclophilin*. Units on the horizontal axes indicate the days after irradiation. Letters a–g indicate significant differences (*p* < 0.05).

Expression patterns of phenylpropanoid and flavonoid biosynthetic genes in bitter melon were investigated at 7, 14, and 21 days under white, blue, or red LED illumination, using real time-PCR (Figure 4). Under white light, the expression of *McC4H*, *McCOMT*, *McFLS*, *McDFR,* and *Mc3GT* increased gradually from 7 to 21 days, whereas that of *McPAL* and *McCHI* transcripts reached a peak level at 14 days, and then declined. The expression of *Mc4CL* and *McCHS* was recovered at 21 days after a decrease at 14 days. Expression of *McF3H* did not change significantly under white light illumination. Under red light conditions, the expression of many phenylpropanoid and flavonoid genes, including *McPAL, Mc4CL, McCOMT, McCHS, McCHI, McF3H, McFLS*, and *Mc3GT,* gradually decreased from 7 to 21 days, except for *McC4H*. Similarly, buckwheat seedlings exposed to red light showed a reduced expression of most orthologous genes after two days of irradiation [27]. The expression of *McDFR* recovered at 21 days after a decrease at 14 days under red light illumination. Compared with the effects of white and red light illumination, bitter melon seedlings exposed to blue light showed greater changes in the expression of phenylpropanoid and flavonoid biosynthetic genes. Expression of *McPAL, McC4H, Mc4CL, McCHS, McCHI, McF3H, McFLS*, *McDFR,* and *Mc3GT* was considerably upregulated compared with seedlings grown under white or red light. Under blue light conditions, the expression of *McC4H, Mc4CL, McCOMT, McCHS, McF3H, McFLS*, *McDFR,* and *Mc3GT* was high at seven days, decreased at 14 days, and then increased again at 21 days. Expression of *McCHI* increased dramatically at 21 days under blue light illumination. However, blue light irradiation had no effect on the expression of *McPAL*.

### 2.6. Effects of White, Blue, and Red Lights on Phenylpropanoid and Flavonoid Content

The contents of phenolic acids, including caffeic acid, rutin, and kaempferol, were measured in seedlings grown under white, blue, or red light for 7, 14, and 21 days (Table 5). Under all light conditions, the accumulation of rutin was the highest (up to 493.96, 433.9, and 87.47 μg/g dry weight under blue, white, and red light irradiation, respectively), followed by that of caffeic acid and kaempferol. However, the effects of white, blue, or red light illumination on caffeic acid, rutin, and kaempferol content varied. Under white light illumination, caffeic acid content gradually increased from 7 to 21 days. In contrast, the rutin content decreased from 433.9 μg/g dry weight at seven days to 195.62 μg/g dry weight at 21 days, whereas kaempferol content reached a peak level at 14 days, and then declined. Under red light illumination, rutin and kaempferol content decreased by about 3.8-fold and 1.8-fold, respectively, whereas caffeic acid content increased by about 2-fold under red light irradiation. This result was congruent with the high expression of *McPAL, Mc4LC, McCHI, McCHS, McF3H, McFLS*, and *Mc3GT* at initial stages, followed by a decrease, under red light irradiation. Kaempferol content remained unchanged under blue light irradiation. The highest rutin and caffeic acid content (493.96.54 and 36.36 μg/g dry weight, respectively) was observed at 21 days under blue light. This highest accumulation of rutin and caffeic acid under blue light irradiation coincided with the increased expression of *McC4H, McCHS, McCHI, McF3H, McDFR,* and *Mc3GT*, suggesting that blue light may induce the phelynpropanoid and flavoloid biosynthetic pathways, leading to increased rutin content in bitter melon. Many previous studies have shown that blue light illumination can affect the accumulation of phenolic compounds in plants. For example, blue light irradiation was effective in increasing the concentration of most phenolic compounds, including *p*-hydroxybenzoic acid, ferulic acid, quercetin, and kaempferol in Chinese cabbage [28]. Blue light caused a high accumulation of polyphenol content in lettuce [24]. A study by Ki-Ho Son and Myung-Min Oh [25] indicated that the ratio of blue to red LEDs is important for the morphology, growth, and the concentration of phenolic compounds with antioxidant properties in green and red leaf lettuce cultivars. In this study, our analysis established that blue light had the optimum wavelength for phenylpropanoid and flavonoid biosynthesis in bitter melon. However, additional research is required to obtain a more comprehensive assessment.

In summary, we identify ten putative biosynthetic enzymes in *Momordica charantia* that have high homology to enzymes involved in the biosynthetic pathway leading to phenylpropanoid and flavonoid synthesis in other plants. Most genes were highly expressed in leaves and/or flowers. An HPLC analysis showed that rutin and epicatechin were most abundant in bitter melon. The accumulation of rutin was highest in leaves, while epicatechin was high accumulated in flowers and fruits. The accumulation patterns of rutin and epicatechin coincide with the expression pattern of *McC4H, McFLS,* and *Mc3GT, and McPAL, Mc4CL, McCOMT, McCHS, McCHI*, *McF3H*, and *McDFR*, respectively, indicating that these genes play important roles in phenylpropanoid and flavonoid biosynthesis in bitter melon. In contrast, the accumulation patterns of benzoic acid did not coincide with the expression pattern of phenylpropanoid and flavonoid biosynthetic genes in *Momordica charantia*. Other phenylpropanoid and flavonoid biosynthetic genes may be linked to the phenylpropanoid and flavonoid synthetic pathway. In addition, we also investigated optimum white, blue, and red light conditions for enhancing phenylpropanoid and flavonoid biosynthesis and found that blue light was the most effective wavelength to enhance accumulation of phenylpropanoid and flavonoid in bitter melon. These results will be of value for developing strategies to increase the yield of medicinal compounds in bitter melon.

## 3. Material and Methods

### 3.1. Plant Materials

**Figure 5:**
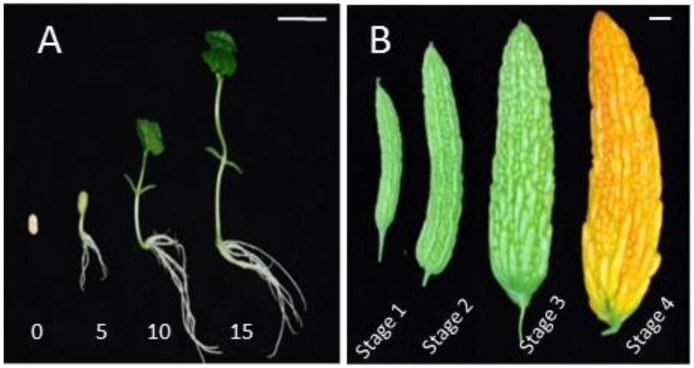
Seedling at 0, 5, 10, and 15 days (**A**), and four developmental stages fruit (**B**) of bitter melon. The scale bars represent 2 cm.

Bitter melon (*Momordica charantia*) seeds were purchased from Beijing Namo Tech.-Trade Co. Ltd. (Beijing, China). After peeling, the seeds were surface-sterilized with 70% (*v*/*v*) ethanol for 30 s and 1% (*v*/*v*) sodium hypochlorite solution for 10 min. The sterilized seeds were germinated using 1/2 MS medium as we described previously [32]. After sowing for 0, 5, 10, and 15 days (Figure 5A and Table S1), the plantlets were harvested. For an LED irradiation experiment, after seeding in a growth chamber at 25 °C for 10 days, bitter melon plantlets were transplanted under white (380 nm), blue (470 nm), or red (660 nm) LED irradiation conditions at the Precision Agriculture Lab, Chungnam National University (Daejeon, Korea). They were grown in a plant factory with the same growth conditions of temperature (25 ± 1 °C), humidity (85–90%), electrical conductivity (1200 ± 90 µS/cm), CO_2_ (1000 ± 100 ppm), light intensity (80–90 µmolm^−2^s^−1^), and photoperiod (16-h light/8-h dark) under different LED irradiation treatments over a 21-day period. Whole plantlets were harvested at 7, 14, and 21 days after irradiation (DAI). In addition, bitter melon was grown at an experimental farm at Chungnam National University (Daejeon, Korea) for three months, for harvesting the plant parts (roots, stems, mature leaves, young leaves, male flowers, and female flowers) and fruits at four different stages (Figure 5B and Table S2). Whole cultivations and irradiations were repeated three times. All samples after harvest were immediately frozen in liquid nitrogen and used for RNA isolation and/or high-performance liquid chromatography-ultraviolet (HPLC-UV) analysis.

### 3.2. RNA Isolation and cDNA Synthesis

Total RNA was isolated from the different plant parts using Easy BLUE Total RNA Kit (iNtRON, Seongnam, Korea), and the quality was confirmed by running a 1.2% agarose gel at 100 W power. ReverTra Ace-kit (Toyobo Co. Ltd, Osaka, Japan) procured from Japan was used for the synthesis of cDNA from 1 μg of total RNA. The prepared cDNA was then diluted 20 times prior to performing quantitative real-time PCR.

### 3.3. Quantitative Real-Time PCR Analysis

The primers of 10 genes involved in the phenylpropanoid and flavonoid pathways were designed using the Primer3 website [33] (details provided in Table S3). For qRT-PCR, the reaction mixture (20 µL) contained primers (0.5 µM), diluted cDNA (10-fold), PCR mixture of 2X SYBR Green (10 µL), and water (5 µL). The PCR machine was run for 3 min (95 °C), 15 s (95 °C, 41 cycles), 15 s (56 °C), and for a final extension of 20 s (72 °C). The *cyclophilin* gene (Accession number: HQ171897.1) was used as the positive gene reference for quantifying the expression of the selected genes. The PCR products were analyzed using the Bio-Rad CFX Manager 2.0 software (Bio-Rad Laboratories, Hercules, CA, USA). The analysis was repeated three times.

### 3.4. High Performance Liquid Chromatography (HPLC) Analysis

Phenylpropanoid and flavonoid compounds were quantified using an HPLC connected to a C18 column (250 mm × 4.6 mm, 5 μm). The samples were mixed with 80% (*v*/*v*) ethanol and incubated at 25 °C for 60 min to complete the extraction of compounds. The samples were eluted in the HPLC system with an elution buffer consisting of methanol and water: acetic acid (98.5:1.5 *v*/*v*) at 1 mL/min^−1^. The levels of phenylpropanoid and flavonoid compounds were calculated based on the peak area of the standard compounds and the calibration curve. Quantification and analysis were performed in triplicate.

### 3.5. Statistical Analysis

Statistical analysis was performed with Statistical Analysis System (SAS version 9.2, SAS Institute Inc., Cary, NC, USA, 2009) using an analysis of variance (ANOVA) with Duncan’s honestly significant difference test. All data are the mean values and standard deviation of triplicate experiments.

## 4. Conclusions

This study provides the first description of the correlation between the putative phenylpropanoid and flavonoid biosynthetic genes and the altered phenylpropanoid and flavonoid contents found in different organs and plantlets of bitter melon. We identified and isolated the cDNAs for 10 genes, including *McPAL, McC4H, Mc4CL, McCOMT, McCHS, McCHI, McF3H, McFLS, McDFR*, and *Mc3GT*, which encoded the phenylpropanoid and flavonoid biosynthetic genes in bitter melon. Most genes were highly expressed in leaves and/or flowers. The accumulation patterns of phenylpropanoid and flavonoid coincided with the expression pattern of *McPAL, McC4H, McCOMT, McFLS,* and *Mc3GT,* indicating that these genes play important roles in phenylpropanoid and flavonoid biosynthesis in bitter melon. In addition, we also investigated white, blue, and red light conditions with respect to their effect on the accumulation of phenylpropanoids and flavonoids in bitter melon. The results of this study demonstrate that blue light is an effective light source for the enhancement of phenylpropanoid and flavonoid biosynthesis. These results will be of value for developing strategies to increase the yield of medicinal compounds in bitter melon.

## Figures and Tables

**Table 1:** Comparison of phenylpropanoid and flavonoid biosynthetic genes of bitter melon with the most orthologous genes.

Genes	Description	Orthologous Genes	Identity (%)
*McPAL* (416 aa)	Phenylalanine ammonia-lyase	*Cucumis sativus* (XP_004145752.1)	91
		*Cucumis melo* (CAA53733.1)	88
		*Vitis vinifera* (XP_002285277.1)	87
*McC4H* (502 aa)	Cinnamate 4-hydroxylase	*Cucumis sativus* (XP_004151467.1)	89
		*Cucumis melo* (XP_008457287.1)	89
		*Salvia miltiorrhiza* (ABC75596.1)	83
*Mc4CL* (556 aa)	4-Coumaroyl CoA ligase	*Cucumis sativus* (XP_004145532.1)	88
		*Cucumis melo* (XP_008452936.1)	87
		*Ziziphus jujuba* (XP_015884135.1)	77
*McCOMT* (369 aa)	Caffeic acid 3-*O*-methyltransferase	*Ricinus communis* (XP_002525818.1)	81
		*Ziziphus jujuba* (XP_015878697.1)	81
		*Prunus mume* (XP_008234634.1)	82
*McCHS* (402 aa)	Chalcone synthase	*Siraitia grosvenorii* (ADF57184.1)	92
		*Cucumis sativus* (XP_004145707.1)	91
		*Cucumis melo* (XP_008449984.1)	92
*McCHI* (252 aa)	Chalcone isomerase	*Cucumis melo* (XP_008456952.1)	86
		*Cucumis sativus* (XP_011655047.1)	86
		*Ricinus communis* (XP_002520870.2)	73
*McF3H* (370 aa)	Flavanone 3-hydroxylase	*Nierembergia sp.* NB17 (BAC10996.1)	81
		*Hibiscus sabdariffa* (ALB35017.1)	86
		*Litchi chinensis* (ADO95201.1)	81
*McFLS* (335 aa)	Flavonol synthase	*Cucumis melo* (XP_008445671.1)	85
		*Cucumis sativus* (XP_004140862.1)	84
		*Theobroma cacao* (XP_007018993.1)	76
*McDFR* (310 aa)	Dihydroflavonol 4-reductase	*Cucumis sativus* (XP_011650548.1)	78
		*Cucumis melo* (XP_008452320.1)	78
		*Cucumis melo* (XP_008452321.1)	78
*Mc3GT* (462 aa)	Flavonoid 3-*O*-glucosyltransferase	*Cucumis sativus* (XP_004140708.1)	89
		*Cucumis melo* (XP_008456146.1)	88

aa: amino acid.

**Table 2:** Accumulation of phenolic compounds in different organs of bitter melon plants (μg/g dry weight).

Compound	Roots	Stems	Y-leaves	M-leaves	F-flowers	M-flowers
Gallic acid	0 ^b^	0 ^b^	0 ^b^	0 ^b^	38.34 ± 9.12 ^a^	0 ^b^
4-Hydroxybenzoic acid	0 ^c^	0 ^c^	29.49 ± 2.72 ^a^	10.86 ± 2.93 ^b^	2.85 ± 1.08 ^c^	0 ^c^
Catechin hydrate	0 ^b^	0 ^b^	0 ^b^	0 ^b^	239.06 ± 19.58 ^a^	0 ^b^
Chlorogenic acid	37.425 ± 0.83 ^c^	40.62 ± 0.90 ^c^	0 ^d^	0 ^d^	49.39 ± 0.57 ^b^	58.7 ± 6.39 ^a^
Caffeic acid	14.43 ± 1.02 ^c^	12.01 ± 0.84 ^c,d^	33.36 ± 2.19 ^a^	24.18 ± 3.27 ^b^	10.17 ± 0.04 ^d^	9.54 ± 2.26 ^d^
Epicatechin	0 ^c^	0 ^c^	0 ^c^	0 ^c^	568.67 ± 12.54 ^b^	660.35 ± 85.84 ^a^
*p*-Coumaric acid	7.65 ± 0.98 ^e^	12.54 ± 0.16 ^d^	54.68 ± 2.66 ^a^	38.07 ± 1.36 ^b^	19.47 ± 0.34 ^c^	10.25 ± 2.94 ^d,e^
Ferulic acid	0 ^c^	4.76 ± 0.26 ^b^	4.8 ± 0.26 ^b^	6.105 ± 1.80 ^b^	9.49 ± 0.23 ^a^	10.27 ± 2.25 ^a^
Benzoic acid	7.19 ± 1.51 ^b^	15.4 ± 4.52 ^a^	0 ^c^	2.85 ± 0.08 ^b,c^	0 ^c^	1.59 ± 0.92 ^c^
Rutin	37.04 ± 2.48 ^d^	153.48 ± 3.12 ^c^	3970.83 ± 27.27 ^a^	2277.93 ± 71.62 ^b^	204.19 ± 28.28 ^c^	7.17 ± 2.76 ^d^
*trans*-Cinnamic acid	0 ^b^	0 ^b^	0 ^b^	0 ^b^	0 ^b^	9.13 ± 2.28 ^a^
Kaempferol	0 ^d^	0 ^d^	1.89 ± 0.00 ^c^	5.49 ± 0.13 ^a^	4.67 ± 0.52 ^b^	0 ^d^

The roots, stems, Y-leaves (young leave), M-leaves (mature leaves), F-flowers (female flowers), M-flowers (male flowers), and four fruit stages (stage 1–4) of 3-month-old plants were collected and used for HPLC analysis. Values are expressed as means ± SD (standard deviation) from three independent measurements. Letters a–e indicate significant differences (*p* < 0.05).

**Table 3:** Accumulation of phenolic compounds in the four fruit stages of bitter melon (μg/g dry weight).

Compound	Stage 1	Stage 2	Stage 3	Stage 4
Chlorogenic acid	37.89 ± 0.03 ^b^	37.74 ± 0.82 ^b^	40.39 ± 1.31 ^a^	40.47 ± 1.65 ^a^
Caffeic acid	13.91 ± 1.66 ^a^	0 ^b^	0 ^b^	0 ^b^
Epicatechin	380.71 ± 10.15 ^a^	386.9 ± 4.75 ^a^	333.22 ± 2.34 ^b^	316.22 ± 9.27 ^c^
*p*-Coumaric acid	0.98 ± 0.43 ^b^	3.02 ± 0.52 ^a^	0.94 ± 0.08 ^b^	1.34 ± 0.44 ^b^
Ferulic acid	4.74 ± 0.00 ^a^	4.79 ± 0.96 ^a^	4.69 ± 0.02 ^a^	4.73 ± 0.06 ^a^
Rutin	12.49 ± 1.09 ^b,c^	13.66 ± 0.68 ^b^	20.7 ± 0.29 ^a^	11.15 ± 0.02 ^c^
Kaempferol	2.66 ± 0.60 ^a^	0 ^b^	0 ^b^	2.72 ± 0.66 ^a^

Values are expressed as means ± standard deviation from three independent measurements. Letters a–c indicate significant differences (*p* < 0.05).

**Table 4:** Accumulation of phenylpropanoid and flavonoid compounds in different developmental stages of bitter melon plantlets (μg/g dry weight).

Compound	0 DAS	5 DAS	10 DAS	15 DAS
4-Hydroxybenzoic acid	0.375 ± 0.15 ^c^	0 ^c^	40.98 ± 1.19 ^a^	18.82 ± 0.54 ^b^
Catechin hydrate	26.52 ± 2.54 ^a^	28.875 ± 0.62 ^a^	13.935 ± 0.40 ^b^	13.12 ± 1.49 ^b^
Chlorogenic acid	34.455 ± 0.23 ^a^	0 ± 0 ^c^	33.225 ± 0.15 ^b^	33.25 ± 0.08 ^b^
Caffeic acid	6.795 ± 0.06 ^a^	6.585 ± 0.02 ^a^	0 ^b^	6.5 ± 0.21 ^a^
*p*-Coumaric acid	2.19 ± 0.29 ^b^	5.145 ± 0.36 ^a^	0.585 ± 0.15 ^c^	0.71 ± 0.03 ^c^
Ferulic acid	4.965 ± 0.15 ^a^	4.905 ± 0.11 ^a^	5.295 ± 0.28 ^a^	5.2 ± 0.15 ^a^
Benzoic acid	4.14 ± 2.20 ^b^	7.92 ± 0.93 ^a^	4.665 ± 0.91 ^b^	6.93 ± 0.24 ^a,b^
Rutin	12.27 ± 0.00 ^d^	27.72 ± 0.85 ^c^	157.935 ± 0.91 ^b^	208.97 ± 3.35 ^a^
*trans*-Cinnamic acid	4.605 ± 0.40 ^a^	1.74 ± 0.00 ^b^	0.66 ± 0.08 ^c^	0.85 ± 0.14 ^c^

Whole bitter melon plantlets were sampled at 0, 5, 10, and 15 DAS and used for HPLC analysis. Values are expressed as means ± standard deviation from three independent measurements. Letters a–d indicate significant differences (*p* < 0.05). DAS: days after sowing.

**Table 5:** Caffeic acid, rutin, and kaempferol content in bitter melon with white, blue, and red LED (μg/g dry weight).

Light	Days	Caffeic acid	Rutin	Kaempferol	Total
**White**	7 days	11.36 ± 1.11 ^e^	433.9 ± 23.3 ^b^	4.71 ± 1.47 ^b^	449.97 ± 24.82 ^b^
	14 days	14.75 ± 0.58 ^d,e^	103.6 ± 6.20 ^d^	9.93 ± 0.41 ^a^	128.28 ± 7.05 ^d^
	21 days	26.71 ± 0.36 ^b,c^	195.62 ± 1.35 ^c^	3.1 ± 0.05 ^b^	225.43 ± 1.58 ^c^
**Blue**	7 days	14.99 ± 0.50 ^d,e^	457.07 ± 13.17 ^a,b^	4.89 ± 0.59 ^b^	476.95 ± 13.17 ^b^
	14 days	31.47 ± 11.67 ^a,b^	221.39 ± 43.85 ^c^	4.56 ± 0.94 ^b^	257.42 ± 56.16 ^c^
	21 days	36.36 ± 0.89 ^a^	493.96 ± 9.02 ^a^	4.3 ± 0.26 ^b^	534.62 ± 9.13 ^a^
**Red**	7 days	11.1 ± 0.49 ^e^	87.47 ± 25.18 ^d^	8.58 ± 0.67 ^a^	107.15 ± 25.56 ^d^
	14 days	20.94 ± 0.70 ^c,d,e^	22.34 ± 0.75 ^e^	3.67 ± 0.21 ^b^	46.95 ± 0.89 ^e^
	21 days	21.67 ± 0.43 ^c,d^	23.15 ± 4.5 ^e^	4.67 ± 0.32 ^b^	49.49 ± 4.64 ^e^

Samples were harvested after 7, 14, and 21 days of irradiation and used for HPLC analysis. Values were expressed as means ± standard deviation from three independent experiments. Letters a–e indicate significant differences (*p* < 0.05).

## Supplementary Materials

The following are available online, Table S1: The development indicators of plantlet of bitter melon. Data represents mean values ± SE (standard error) of three replicates. DAS—days after sowing, Table S2: The size of four developmental fruit stages of bitter melon. Data represents mean values ± SE of three replicates, Table S3: Sequences of specific primers used for quantitative real-time PCR.

## References

[1] Meng Y., Liu S., Li J., Meng Y., Zhao X. (2012). Preparation of an antitumor and antivirus agent: Chemical modification of alpha-mmc and map30 from *Momordica charantia* L. with covalent conjugation of polyethyelene glycol. Int. J. Nanomed..

[2] Fang E.F., Ng T.B. (2011). Bitter gourd (*Momordica charantia*) is a cornucopia of health: A review of its credited antidiabetic, anti-HIV, and antitumor properties. Curr. Mol. Med..

[3] Tahira S., Hussain F. (2014). Antidiabetic evaluation of *Momordica charantia* L. fruit extracts. West Indian Med. J..

[4] Puri M., Kaur I., Kanwar R.K., Gupta R.C., Chauhan A., Kanwar J.R. (2009). Ribosome inactivating proteins (RIPs) from *Momordica charantia* for antiviral therapy. Curr. Mol. Med..

[5] Kabir S.R., Nabi M.M., Nurujjaman M., Abu Reza M., Alam A.H., Uz Zaman R., Khalid-Bin-Ferdaus K.M., Amin R., Khan M.M., Hossain M.A. (2015). *Momordica charantia* seed lectin: Toxicity, bacterial agglutination and antitumor properties. Appl. Biochem. Biotechnol..

[6] Gurbuz I., Akyuz C., Yesilada E., Sener B. (2000). Anti-ulcerogenic effect of *Momordica charantia* L. fruits on various ulcer models in rats. J. Ethnopharmacol..

[7] Saeed M.K., Shahzadi I., Ahmad I., Ahma R., Shahzad K., Ashraf M., Viqar-un-Nisa A. (2010). Nutritional analysis and antioxidant activity of bitter gourd (*Momordica charantia* L.) from Pakistan. Pharmacologyonline.

[8] Korkina L.G. (2007). Phenylpropanoids as naturally occurring antioxidants: From plant defense to human health. Cell. Mol. Biol..

[9] Castelluccio C., Paganga G., Melikian N., Bolwell G.P., Pridham J., Sampson J., Riceevans C. (1995). Antioxidant potential of intermediates in phenylpropanoid metabolism in higher-plants. FEBS Lett..

[10] Agati G., Azzarello E., Pollastri S., Tattini M. (2012). Flavonoids as antioxidants in plants: Location and functional significance. Plant. Sci..

[11] Li X.H., Il Park N., Xu H., Woo S.H., Park C.H., Park S.U. (2010). Differential expression of flavonoid biosynthesis genes and accumulation of phenolic compounds in common buckwheat (*Fagopyrum esculentum*). J. Agric. Food Chem..

[12] Li X., Kim J.K., Park S.Y., Zhao S., Kim Y.B., Lee S., Park S.U. (2014). Comparative analysis of flavonoids and polar metabolite profiling of tanno-original and tanno-high rutin buckwheat. J. Agric. Food Chem..

[13] Ghimeray A.K., Sharma P., Phoutaxay P., Salitxay T., Woo S.H., Park S.U., Park C.H. (2014). Far infrared irradiation alters total polyphenol, total flavonoid, antioxidant property and quercetin production in tartary buckwheat sprout powder. J. Cereal Sci..

[14] Lee L.S., Choi E.J., Kim C.H., Sung J.M., Kim Y.B., Seo D.H., Choi H.W., Choi Y.S., Kum J.S., Park J.D. (2016). Contribution of flavonoids to the antioxidant properties of common and tartary buckwheat. J. Cereal Sci..

[15] Thwe A.A., Kim J.K., Li X.H., Kim Y.B., Uddin M.R., Kim S.J., Suzuki T., Park N.I., Park S.U. (2013). Metabolomic analysis and phenylpropanoid biosynthesis in hairy root culture of tartary buckwheat cultivars. PLoS ONE.

[16] Park D., Park Y., Lee Y.H., Choi I.Y., Park K.C., Park S.U., Kim B.S., Yeoung Y.R., Park N.I. (2017). A comparative study of phenolic antioxidant activity and flavonoid biosynthesis-related gene expression between summer and winter strawberry cultivars. J. Food Sci..

[17] Zhao S., Park C.H., Li X., Kim Y.B., Yang J., Sung G.B., Park N.I., Kim S., Park S.U. (2015). Accumulation of rutin and betulinic acid and expression of phenylpropanoid and triterpenoid biosynthetic genes in mulberry (*Morus alba* L.). J. Agric. Food Chem..

[18] Kim J.K., Kim Y.S., Kim Y., Uddin M.R., Kim Y.B., Kim H.H., Park S.Y., Lee M.Y., Chung S.O., Park S. (2014). Comparative analysis of flavonoids and polar metabolites from hairy roots of *Scutellaria baicalensis* and *Scutellaria lateriflora*. World J. Microbiol. Biotechnol..

[19] Auyeung K.K., Han Q.B., Ko J.K. (2016). Astragalus membranaceus: A review of its protection against inflammation and gastrointestinal cancers. Am. J. Chin. Med..

[20] Park Y.J., Thwe A.A., Li X.H., Kim Y.J., Kim J.K., Arasu M.V., Al-Dhabi N.A., Park S.U. (2015). Triterpene and flavonoid biosynthesis and metabolic profiling of hairy roots, adventitious roots, and seedling roots of *Astragalus membranaceus*. J. Agric. Food Chem..

[21] Horax R., Hettiarachchy N., Islam S. (2005). Total phenolic contents and phenolic acid constituents in 4 varieties of bitter melons (*Momordica charantia*) and antioxidant activities of their extracts. J. Food Sci..

[22] Massa G.D., Kim H.H., Wheeler R.M., Mitchell C.A. (2008). Plant productivity in response to led lighting. HortScience.

[23] Morrow R.C. (2008). Led lighting in horticulture. HortScience.

[24] Johkan M., Shoji K., Goto F., Hashida S., Yoshihara T. (2010). Blue light-emitting diode light irradiation of seedlings improves seedling quality and growth after transplanting in red leaf lettuce. HortScience.

[25] Son K.H., Oh M.M. (2013). Leaf shape, growth, and antioxidant phenolic compounds of two lettuce cultivars grown under various combinations of blue and red light-emitting diodes. HortScience.

[26] Hossen M.Z. (2007). Light emitting diodes increase phenolics of buckwheat (*Fagopyrum esculentum*) sprouts. J. Plant Interact..

[27] Thwe A.A., Kim Y.B., Li X., Seo J.M., Kim S.J., Suzuki T., Chung S.O., Park S.U. (2014). Effects of light-emitting diodes on expression of phenylpropanoid biosynthetic genes and accumulation of phenylpropanoids in *Fagopyrum tataricum* sprouts. J. Agric. Food Chem..

[28] Kim Y.J., Kim Y.B., Li X., Choi S.R., Park S., Park J.S., Lim Y.P., Park S.U. (2015). Accumulation of phenylpropanoids by white, blue, and red light irradiation and their organ-specific distribution in chinese cabbage (*Brassica rapa* ssp. *Pekinensis*). J. Agric. Food Chem..

[29] Cuong D.M., Jeon J., Morgan A.M.A., Kim C., Kim J.K., Lee S.Y., Park S.U. (2017). Accumulation of charantin and expression of triterpenoid biosynthesis genes in bitter melon (*Momordica charantia*). J. Agric. Food Chem..

[30] Zhao S., Tuan P.A., Li X., Kim Y.B., Kim H., Park C.G., Yang J., Li C.H., Park S.U. (2013). Identification of phenylpropanoid biosynthetic genes and phenylpropanoid accumulation by transcriptome analysis of *Lycium chinense*. BMC Genomics..

[31] Li X., Park N.I., Xu H., Woo S.H., Park C.H., Park S.U. (2010). Differential expression of flavonoid biosynthesis genes and accumulation of phenolic compounds in common buckwheat (*Fagopyrum esculentum*). J. Agric. Food Chem..

[32] Murashige T., Skoog F. (1962). A revised medium for rapid growth and bioassays with tobacco tissue cultures. Physiol. Plant..

[33] Untergasser A., Cutcutache I., Koressaar T., Ye J., Faircloth B.C., Remm M., Rozen S.G. (2012). Primer3—new capabilities and interfaces. Nucleic Acids Res..

