# Supplementary material for: Identification and Characterization of Phenylpropanoid Biosynthetic Genes and Their Accumulation in Bitter Melon (Momordica charantia)
Source: Molecules. 2018 Feb 21;23(2):469. doi: 10.3390/molecules23020469 (PMC6016960; doi:10.3390/molecules23020469)
Supplement: Supplementary file 1 [file molecules-23-00469-s001.pdf]

Table S1. The development indicators of plantlet of bitter melon. Data represents mean values ± SE of three replicates. DAS, days after sowing.

	**Fresh weight (g)**	**Stems length (cm)**	**Roots length (cm)**
0 DAS	0.18 ± 0.01	0 ± 0	0 ± 0
5 DAS	0.48 ± 0.05	2.64 ± 0.20	2.17 ± 0.31
10 DAS	2.51 ± 0.14	9.30 ± 0.62	10.17 ± 0.63
15 DAS	3.50 ± 0.21	15.89 ± 1.36	12.61 ± 1.36

Table S2. The size of four developmental stages fruit of bitter melon. Data represents mean values ± SE of three replicates.

**Indicator**	**Stage1**	**Stage2**	**Stage3**	**Stage4**
length (cm)	15 ± 0.5	26 ± 0.4	28 ± 0.2	30 ± 0.3
weight (g)	61 ± 0.7	228 ± 0.9	414 ± 0.6	515 ± 1.3

Table S3. Sequences of specific primers used for quantitative real-time PCR

**Primers name**	**Forward primer sequences (5' to 3')**	**Reverse primer sequences (5' to 3')**	**Size (bp)**
McPAL	ATTGGGAAGCTCATGTTTGC	GGTGACGGGATTTGCTAAGA	177
McC4H	CTTCAACAAGTGGGGTGGTT	GCCTGAGAATTGGGATGAAA	198
Mc4CL	GCCACGTCATCCTCCTTCTA	CGATTTGTTTGGCGATTTCT	132
McCOMT	CTGTTGAAGAATTGCCACGA	GGGTTGTGAGCCAGCATTAT	140
McCHS	GGCTTACGTTTCACCTCCTG	CGTGGCTCTCATTTTGTCCT	197
McCHI	AAGGGGAAACCAGCTAAGGA	CACTCTCCAACTGCACTCCA	139
McF3H	ACGGAGGAGTACAGCGAGAA	AGGGCATTTGGGGTAGAAAT	150
McFLS	CTTCAGAGATGGGCATTGGT	CTTGTCCACCGTCGTCCTAT	130
McDFR	CGTCAATGGATCATGTCTGG	TTGGTGCTGTGAGAACTTGC	195
Mc3GT	GCAGAGCTGGGAAAGATGAC	AGACCGCAGACCTTCAAAGA	188
McCYP	GGCAAACCCTAAAGTTTTCTTCG	GATGAGCCCTTGTAATGAAGTGG	174
